# Individualized monitoring of longitudinal heading exposure in soccer

**DOI:** 10.1038/s41598-024-52163-8

**Published:** 2024-01-20

**Authors:** Rebecca Kenny, Marko Elez, Adam Clansey, Naznin Virji-Babul, Lyndia C. Wu

**Affiliations:** 1 Department of Mechanical Engineering, University of British Columbia, 6250 Applied Science Ln Room 2054, Vancouver, BC V6T 1Z4 Canada; 2 Department of Integrated Sciences, University of British Columbia, 6356 Agricultural Rd Room 464, Vancouver, BC V6T 1Z2 Canada; 3 Department of Physical Therapy, The University of British Columbia, 2177 Wesbrook Mall, Vancouver, BC V6T 1Z3 Canada

**Keywords:** Risk factors, Biomedical engineering

## Abstract

There is growing concern that repetitive soccer headers may have negative long-term consequences on brain health. However, inconsistent and low-quality heading exposure measurements limit past investigations of this effect. Here we conducted a comprehensive heading exposure analysis across all players on a university women’s soccer team for over two calendar years (36 unique athletes), quantifying both game and practice exposure during all in-season and off-season periods, with over ten thousand video-confirmed headers. Despite an average of approximately 2 headers per day, players’ daily exposures ranged from 0 to 45 headers, accumulating to highly variable total exposure of 2–223 headers over each in-season/off-season period. Overall, practices and off-season periods accounted for 70% and 45% of headers, respectively. Impact sensor data showed that heading kinematics fell within a tight distribution, but sensors could not capture full heading exposure due to factors such as compliance. With first-of-its-kind complete heading exposure data, we recommend exposure assessments be made on an individual level and include practice/off-season collection in addition to games and competitive seasons. Commonly used group statistics do not capture highly variable exposures, and individualized complete heading exposure tracking needs to be incorporated in future study designs for confirming the potential brain injury risk associated with soccer heading.

## Introduction

Headers are considered an important skill in soccer whereby players can shoot, pass, or defend when a ball is airborne by intentionally striking the ball with their head^1^. Although it has been noted that heading is necessary to maintain the integrity and competitiveness of soccer^2^, there is growing concern over the potential association between heading and long‐term changes in brain structure and function^3–5^. While ball-to-head impacts rarely cause concussion^5,6^, some studies in both amateur and professional contact sport athletes, including a small number of soccer cases, indicate that repetitive sport impact exposure may be correlated with neurodegenerative pathology associated with chronic traumatic encephalopathy (CTE)^7–9^. While there are studies supporting increased neurodegenerative disease risk in former professional soccer players^5,7^, there is still much debate on the strength of evidence to prove this link. For example, some studies reported decline in memory and attention^8–10^ and cortical thinning in former professional soccer athletes^11^, and others with similar study design had negative findings^3,12–14^. Inconclusive results in the study of brain injury risk may be a result of limitations in the measurement and interpretation of heading exposure. This may also be an area where group-statistics may not be appropriate due to the high individual variance and lack of group-to-individual generalizability^15^.

A systematic review on the relationship between soccer heading and persistent brain changes identified inconsistent and low-quality heading exposure measurements across studies as the greatest limitation in characterizing brain injury risk, indicating accurate estimates of heading frequency are necessary to determine if such correlations exist^4^. A systematic review by McCunn et al. on the incidence of heading found it difficult to reach consensus on actual heading exposure during games and practices due to variations in methodologies and populations studied^16^. Even studies that used a common metric of average headers per player per game reported a wide range of average values between 1 and 9^16^. A 2023 review suggests that heading exposure may not be evenly distributed across a team, and a reliance on solely reporting average values may not fully capture the potential athlete-to-athlete variance^17^. Selecting the most accurate methodologies for collecting and reporting exposure data is therefore necessitated when correlating to long-term outcomes.

There is evidence to suggest that certain data collection methods are more reliable than others. Video analysis is considered the ‘gold standard’ in identifying soccer heading exposure, providing strong interrater and intrarater agreement when classifying and counting headers^18^. The time-consuming and resource-intensive nature of video analysis has limited its use in exposure studies that quantify impacts on a large scale^19^. While studies have primarily focused on games, practices may involve a greater proportion of headers^16,18,20^. In the aforementioned McCunn systematic review, reliance on self-reported exposure was identified as another common limitation in prior studies, and even in the 42 reviewed studies where headers were independently observed, only one study included video analysis of all practices and games over a competitive season of play (our secondary analysis of heading exposure measurement method is included in Supplementary Table 1)^16^. However, even this may not paint a complete picture of heading exposure, since many teams often practice year-round, and an accurate longitudinal exposure estimate may also need to include data outside of the competitive season.

In addition to the number of head impacts a player is exposed to, cumulative head impact exposure as it pertains to long term outcomes has also been defined as a function of impact magnitudes^18^. Wearable head impact sensors can provide measurements of head impact kinematic data^21^. However, issues with cost, accessibility, compliance, and sensor errors such as false positive or false negative detection, may limit the feasibility and scope of such instrumentation, especially for longitudinal studies^19,22–24^. While laboratory studies using mouthguard-based sensors have reported up to 96% sensitivity in recording dummy head impacts, on-field sensitivity may be much lower^25^. An on-field validation study assessing the sensitivity of mouthguard sensors in detecting headers compared to video analysis reported a sensitivity of 69.2% (i.e., over 30% false negatives)^21^. Additionally, mouthguard sensors may identify acceleration events not associated with head impacts, with one on-field validation study reporting nearly 80% of the recordings being false positives^26^. Our recent study also demonstrates potential bias in exposure measurement due to commonly applied acceleration thresholding methods, with simulation showing that even impacts with 30 g peak head centre-of-gravity linear acceleration may not trigger recording at the sensor location^27^. As such, sensor instrumentation, while providing valuable impact kinematics information, often suffer from missing data and video information may still be crucial in providing full longitudinal exposure estimates^28^.

Here we conducted a longitudinal heading exposure study of all athletes on a varsity women’s soccer team including both games and practices during in-season and off-season sessions for over two calendar years. The primary goal of the study was to propose a framework for more accurate quantification of complete longitudinal heading exposure with three main objectives. First, we assessed the level of individual variation in heading exposure among players on the same team to evaluate the effectiveness of previous reporting metrics which describe heading exposure as an average rate. Second, we determined the necessity of including practice data and off-season data in estimating longitudinal heading exposure. Finally, we evaluated variance in header impact severity through contact scenario characterization and a subsample of sensor-measured impacts to inform the effectiveness and necessity of impact sensors for capturing longitudinal heading exposure. Through this work, we aim to promote more rigorous research of long-term heading exposure consequences.

## Methods

### Overview

We conducted a longitudinal observational cohort study to analyse heading exposure of all players from all practices and games in a collegiate women’s soccer team from August 2019 to December 2021. In this study, we divided each calendar year into three periods: in-season, off-season, and summer off-season. These definitions were selected on the basis that team composition remained consistent within a single period, with any roster changes occurring between periods. In-season play coincided with the official varsity league play (August–December); the off-season was a non-optional practice period coinciding with the university calendar (January–April); an optional practice period occurred in the summer off-season (May–August). The COVID-19 pandemic interrupted the 2020–2021 off-season and there was no summer session. 

### Video data and review process

**Figure 1:**
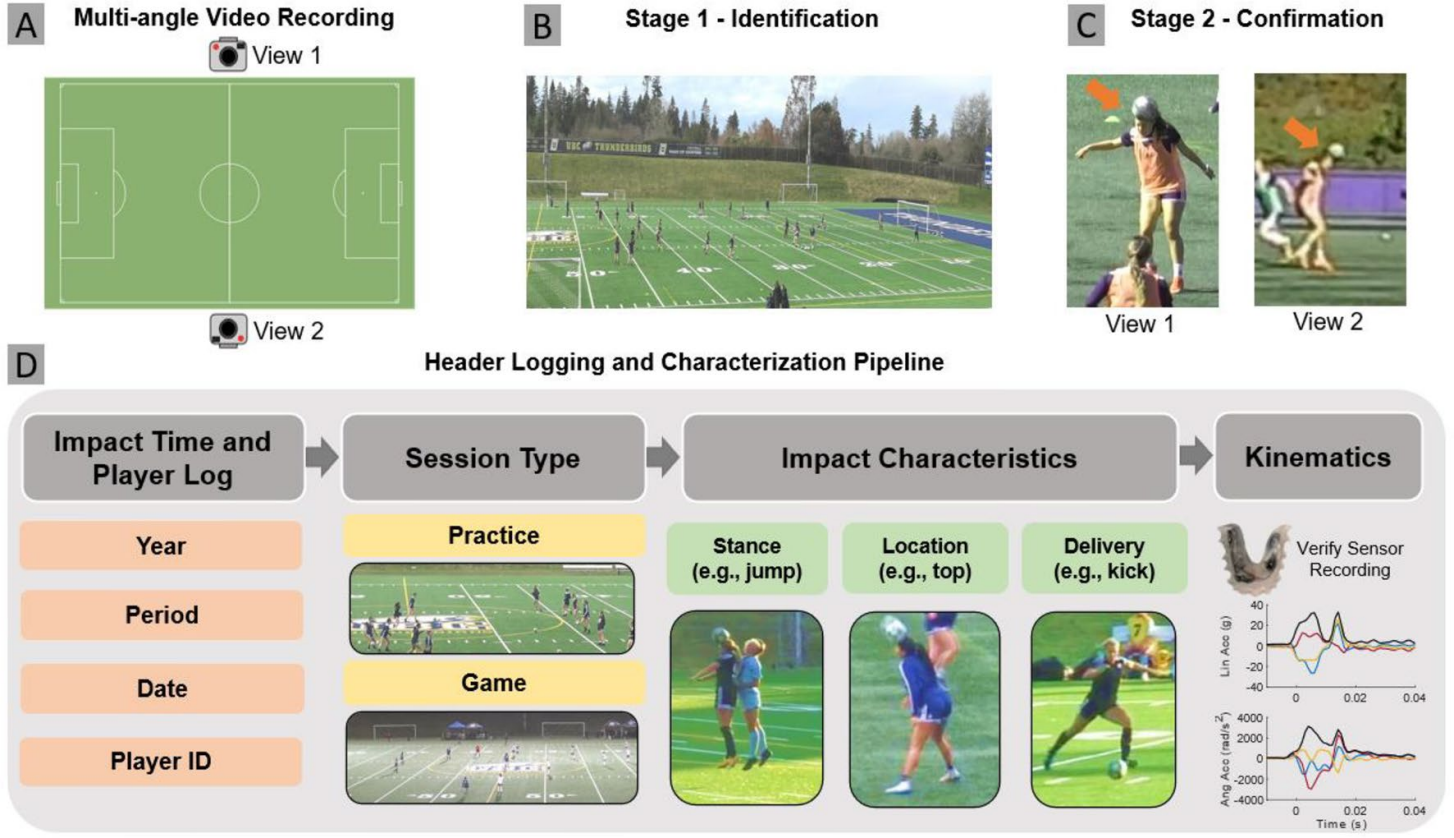
Methodology. For each practice or game, we analyzed video footage from at least two camera views (**A**). We conducted a 2-stage video analysis protocol, where in the first stage, videos were reviewed to identify all potential headers with high sensitivity (**B**), and in the second stage, both views were reviewed to confirm headers (**C**). Panel (**D**) summarizes the video analysis pipeline used to categorize each header.

Figure 1 illustrates our study methodology. We used video footage with either 2.7 K or 4 K resolution and 60 fps frame rate from each practice and game (Fig. 1A,B). Video was captured using four angles in practices and home games, and two angles during away games. For practices and home games, two cameras were set up with a 10 m elevation in line with the center of the field, and two other cameras were positioned in the far corners of the opposite side of the field at 1.5 m elevation. The 2019 in-season period and some home games were exceptions where only two 10 m elevation cameras were used, due to an initial trial period with limited camera equipment and the team’s requests to minimize interference with game activity, respectively. A second-resolution clock was used to synchronize multiple cameras. We applied a two-stage video review process using the *PotPlayer* video player. In stage 1, 16 research assistants were instructed to identify any potential ball-to-head contact events in video, minimizing subjective judgement and maximizing sensitivity of detection (Fig. 1B). Research assistants were first trained by reviewing video footage of a game with the number of headers (n = 41) identified and verified by an expert reviewer. The sensitivity of all reviewers was confirmed to be 100% for capturing verified headers in the training video. Since ball-to-head contact is a clearly identifiable video event, we expected a high level of sensitivity in header detection through video. In stage 2, using slow-motion or frame-by-frame video playback, lead author RK verified and characterized each potential header by reviewing multi-angle information and examining if ball-to-head contact could be confidently identified where a ball trajectory deflection occurred due to contact (Fig. 1C). We estimate that the time burden associated with our video analysis was a median of 2.5 h of review time per hour of video, for a total of over 1200 reviewer-hours spent for the current study. Two games during the Fall 2019 season and one game during the Spring 2020 season were excluded due to poor video quality.

### Video analysis methods

Each ball-to-head impact was categorized based on when the impact occurred, player position (forward, midfield, defender), on-field activity (practice drill, scrimmage, game), player stance (jumping, not jumping), head impact location (forehead/top/left/right/back) and ball delivery method (long kick, short kick, throws) (Fig. 1D). A detailed explanation of the impact characteristics or contact scenario can be found in a prior publication^29^. We divided activity types into practices and games, and further divided practices into drills and scrimmages (see Supplemental Information for a breakdown of each activity type). To calculate commonly used average exposure rates, heading exposure was defined in terms of headers per hour of play as well as headers per day. Time on field was calculated per player by determining the total number of on-field minutes for a given practice and by monitoring game or scrimmage substitutions. Headers per day or per hour were calculated by dividing the total number of headers by the number of days and total hours of on-field play respectively.

### Kinematic data collection

A sensor sub-study was conducted from September 2020 to December 2021, with a varying subset of players (total = 13) consenting to be instrumented with the Wake Forest University mouthpiece sensor^21^. Firmware version 0.5.1 was loaded onto the mouthpieces. Mouthpieces contain a tri-axis accelerometer with a 5 g per-axis linear acceleration threshold, to measure linear acceleration and angular velocity. Further details on the sensor methods can be found in a previous publication^29^. Whereas video-verified head impacts are confirmed by identifying ball-to-head contact during video review, sensor-identified impacts are detected by impact sensors based on sensor-measured kinematics passing a pre-set trigger threshold. Only sensor impacts that match with video-verified headers were included for kinematic data analysis. To examine patterns in kinematics variability in linear and angular accelerations over time and between players, we selected a subset of data from the study period with the most video verified headers captured by sensors (2021 off-season). Our selection criterion for players was > 70% video-verified headers captured by sensors (n = 4). No other athletes had device sensitivity (i.e., capture rate of video-verified headers) of greater than 50% due to a combination of factors including sensor malfunction, noncompliance, and false negative detections.

### Data analysis and statistics

To address our study objectives, we quantified per-session heading exposure and total heading exposure over each in-season/off-season/summer off-season period for each individual player. We analyzed the individual variance across each period, per activity type and per player to determine if average team metrics could accurately represent heading exposure. We compared practice exposures to game exposures as well as off-season/summer off-season exposures to in-season exposure to determine if relying on games and in-season exposure accurately represents overall exposure. Since heading exposures followed highly skewed distributions, we report descriptive statistics using median, interquartile range (IQR), and absolute range (AR). In addition, we compared practice drills, scrimmages and games in their prevalence of contact scenarios with higher header impact kinematics. These scenarios of interest include long kicks, top-of-head heading impacts, and jumping heading impacts^29^. To examine the feasibility of using sensors to capture full heading exposure, we quantified sensor failure rates, player compliance rates, and device sensitivity for all instrumented players across the full length of our sensor sub-study. Heading impact kinematics experienced over time and distributions of peak linear accelerations (PLA) and peak angular accelerations (PAA) were examined for the four individual players meeting the selection criterion included in the study.

### Ethics approval and consent to participate

The study protocol was approved by the University of British Columbia Research Ethics Board (Protocol H17-02973). The research was performed in accordance with the Declaration of Helsinki, and informed consent was obtained from all participants that participated in wearable sensor measurements of exposure. Individual consent was determined by the UBC Research Ethics Board to be exempt for athletes whose exposure was only observed through team videos, with written permission from the team coach.

## Results

**Figure 2:**
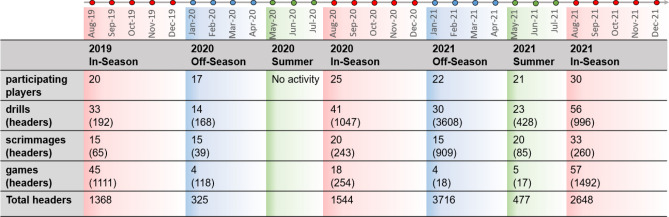
Data overview. We analyzed data from August 2019 to December 2021, with a gap from April 2020 to June 2020 due to the COVID-19 pandemic. This figure shows a breakdown of the number of participating players, the number of drills/scrimmages/games, the number of headers per activity type, and the total headers per period.

Our study includes data from all players on a university varsity women’s soccer team from August 2019 to December 2021. During this time, 36 unique players participated at varying times, with 17–30 players per period, 14 of whom participated in all periods. Video data were collected during 263 days (129 games and 208 practices) accounting for a total of 7753 athlete-hours and 4947 athlete-days when summed across the entire team. We identified and analyzed a total of 10,078 headers (Fig. 2). We conducted kinematic data analysis using sensor data from four players for a total of 578 headers (15.5% of headers) and 137 athlete-days (18.7% of all athlete-days) over the 2021 off-season period.

### Individual variability in heading exposure

First, we evaluated player-to-player variability in longitudinal and daily heading exposures. Using commonly applied average metrics, we observed an average rate of headers across all activity types of 1.3 headers per hour and 2.04 headers per day. Table 1 illustrates a large range in the number of headers experienced by players across an entire period and on individual days. A player’s total heading exposure in a period ranged from 2 to 223 headers. While the summer period had the lowest median heading exposure, it contained the highest individual exposure (223); and although regular season median exposure was the highest, similarly wide ranges of exposures were observed in both the off-season and summer periods. Aside from the cumulative exposure variance, daily exposures can also be highly variable for a player. The majority of players did not sustain any headers on most activity days across all types of periods. The percentage of sessions with zero exposure for each session type can be found in our Supplemental Data. On days with exposure, a player experienced between 1 and 45 heading impacts, showing that some daily exposures could surpass those experienced over a 3–5 month period.

**Table 1:** Heading exposure variability across players.

	Regular season Median (IQR, AR)	Off-season period Median (IQR, AR)	Summer period Median (IQR, AR)
Summary of cumulative exposure			
Total number of headers sustained in the period by a player	172.5 (82–208, 26–209)	36 (3.5–167.3, 2–209)	7.5 (4–18, 2–223)
Percentage of headers sustained during practice by a player	54.5% (36.6–70.5%, 7.7–100%)	97.5% (92.2–99.5%, 50.0–100%)	81.0% (73.9–95.3%, 25–100%)
Summary of per-day exposure			
Percentage days with zero headers for a player	74.1% (67.7–77.2%, 56.2–85.1%)	66.2% (58.3–74.2%, 50–100%)	68.7% (61.0–80.0%, 45.4–82.6%)
Number of headers per day for a player on days with exposure	2 (1–3, 1–34)	3 (1–8, 1–45)	1 (1–2, 1–36)

*IQR* interquartile range, *AR* absolute range.

**Figure 3:**
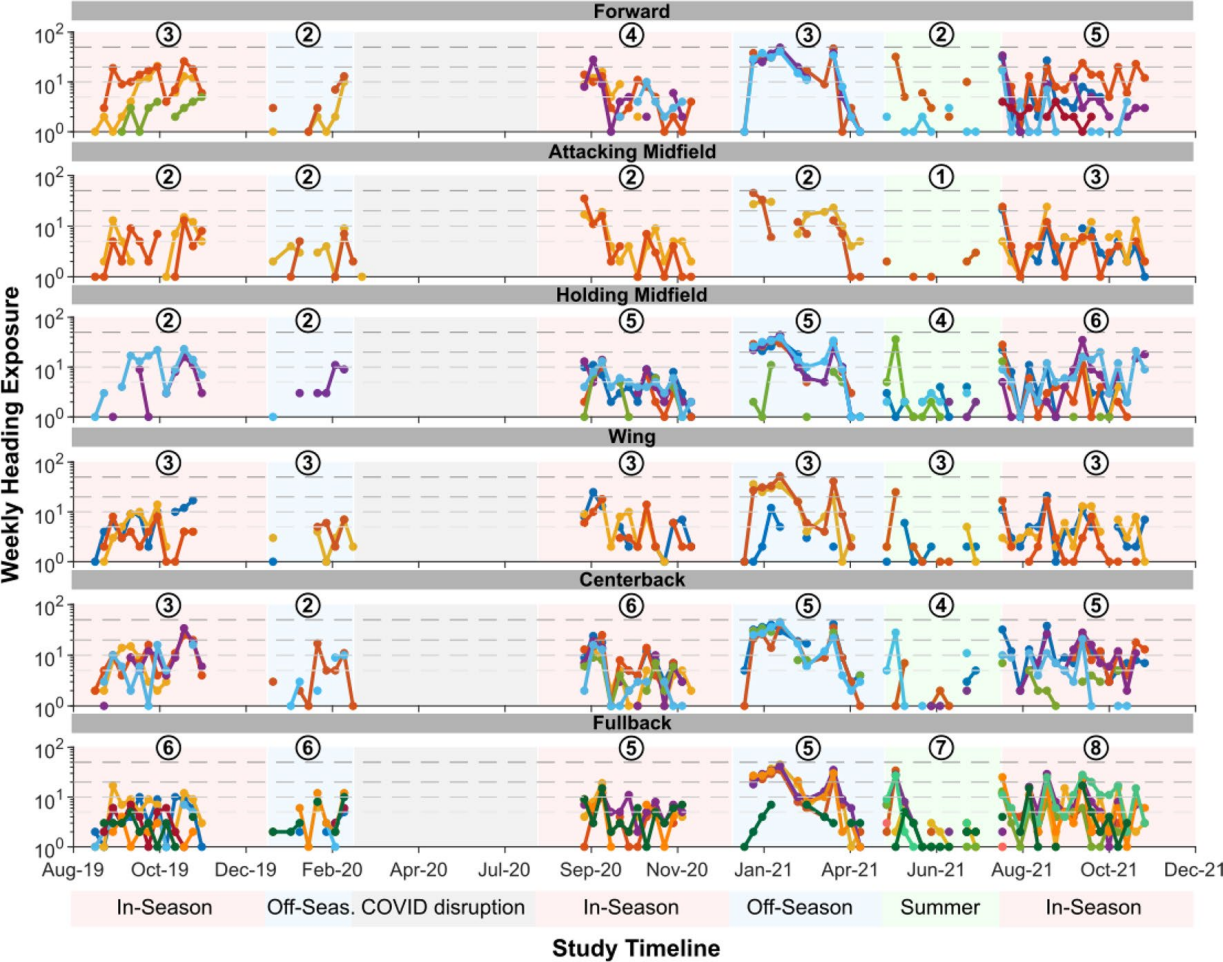
Variability in longitudinal heading exposure of individual players. Here we show a comparison of heading exposure across all players and position groups over the entire study duration. Each player is represented by a differently coloured line in each position group. Each data point represents the total number of headers experienced by a single player in 1 week. The circled numbers above each period indicate the number of players studied in that period for that position group. Only non-zero weekly exposures are shown in this figure.

To further examine exposure variability over time, we illustrate the heading exposure timelines of all players followed in the current study, organized by position group (Fig. 3). Each player in a given position group is denoted by a differently coloured line, where the y-axis indicates the number of headers experienced by each player in a single week. This figure demonstrates high variability in individual exposure over the longitudinal study. Variability exists both within and across individuals, and there does not seem to be position group-specific patterns. While some weeks may have more consistently high exposure for most players (e.g., weeks between Jan 2021 and Mar 2021), other weekly exposures can be more variable, making it difficult to extract consistent exposure patterns over time even for the same position group on the same team.

### Practice and off-season exposure

**Figure 4:**
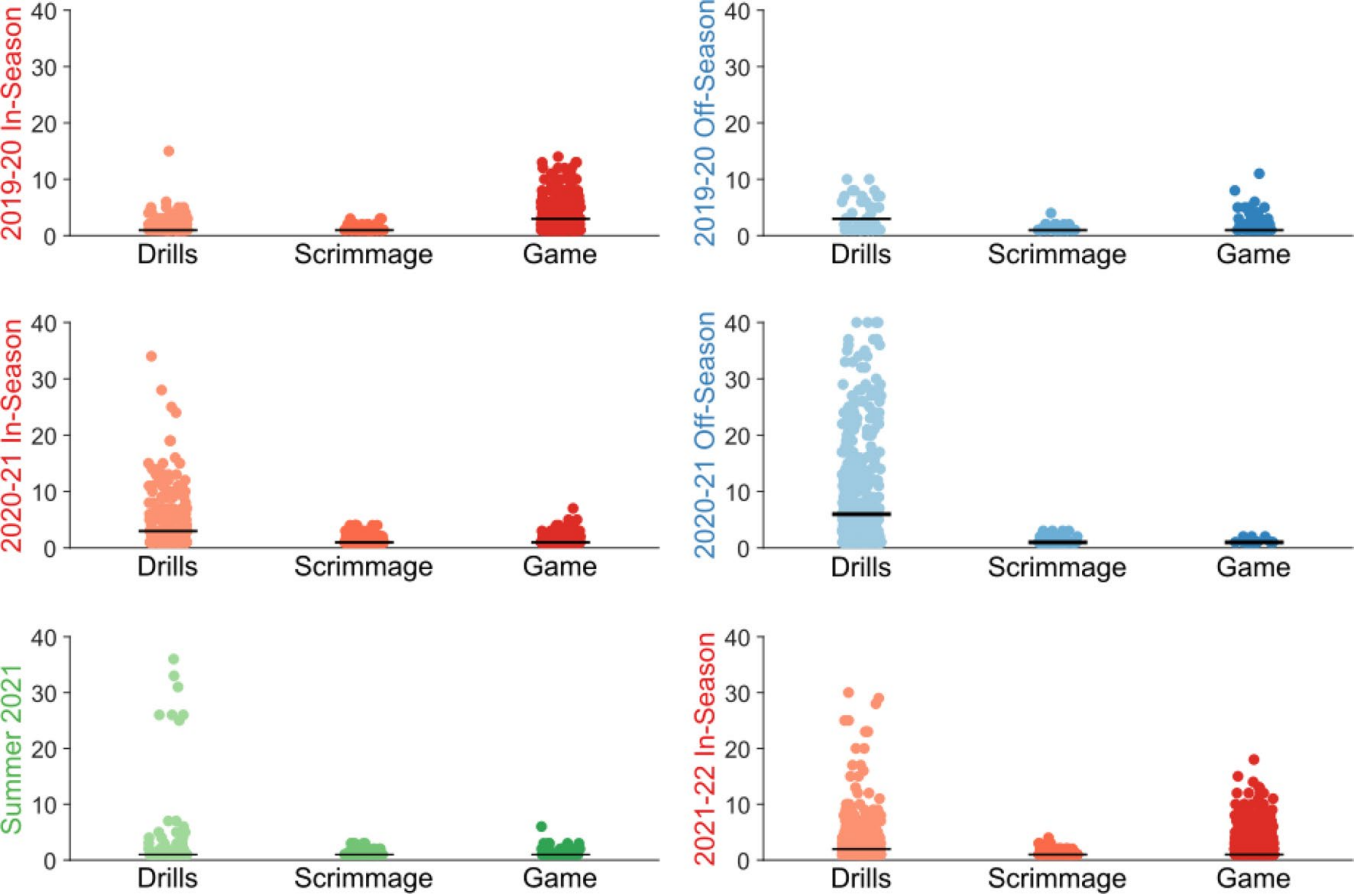
Distribution of per-session heading exposure across activity types and periods. Here we illustrate the variance in distribution of headers in different activity types (drill, scrimmage, game) across different in-season/off-season/summer periods. Each point represents a single player’s exposure during a session. Black bars represent the median exposure level calculated from sessions with non-zero heading exposure, since players have substantial numbers of zero-header sessions. The percentage of sessions with zero headers are detailed in Supplementary Table 2.

Practices accounted for substantial proportions of players’ heading exposure. Among all the headers we identified, 7019 headers occurred during practices (70%), compared to 3059 headers during games (30%). For practices, 6337 headers (90%) occurred during drills, and 682 headers (10%) occurred during scrimmages. As shown in Table 1, practices can account for the majority of a player’s heading exposure during a calendar year. Even during the regular season, each player sustained a median of 54.5% of their heading exposure during practices. While game-day heading exposures had a maximum of 18 headers, practices showed highly variable exposures up to over 40 headers experienced on a single day, with the majority of exposure from drills (Fig. 4). In five out of six periods studied, the highest number of headers for a single player in a single day occurred during practice drills.

Overall, we recorded 5559 (55%) headers during three in-season periods, 4043 (40%) during two off-season periods and 477 (5%) for one summer period. The most headers (3718) occurred during the 2020–2021 off-season. For the 14 players that participated in the full study duration, a median of 44.0% headers were sustained during off-season periods (IQR 39.6–51.8%, AR 1.3–61.8%). As shown in Table 1, the summer off-season period included greatest exposure for an individual player in a single period (223 headers), while the median exposure was only 7.5 headers for the period. Median individual heading exposure per day (3 headers/day) was greatest during the off-season periods.

### Impact magnitude

The contact scenarios associated with higher heading impact magnitudes commonly occurred not only during games, but also during drills and scrimmages. During drills, we observed that 22%, 41%, and 13% of headers resulted from jump, top-of-head, and long kick scenarios, respectively. During scrimmages, we observed 59%, 52%, and 64% headers to be from jump, top-of-head, and long kick scenarios, respectively. Practice drills and games differ greatly in the most common stances, head locations, and ball deliveries that occur, while scrimmages and games are more similar.

**Figure 5:**
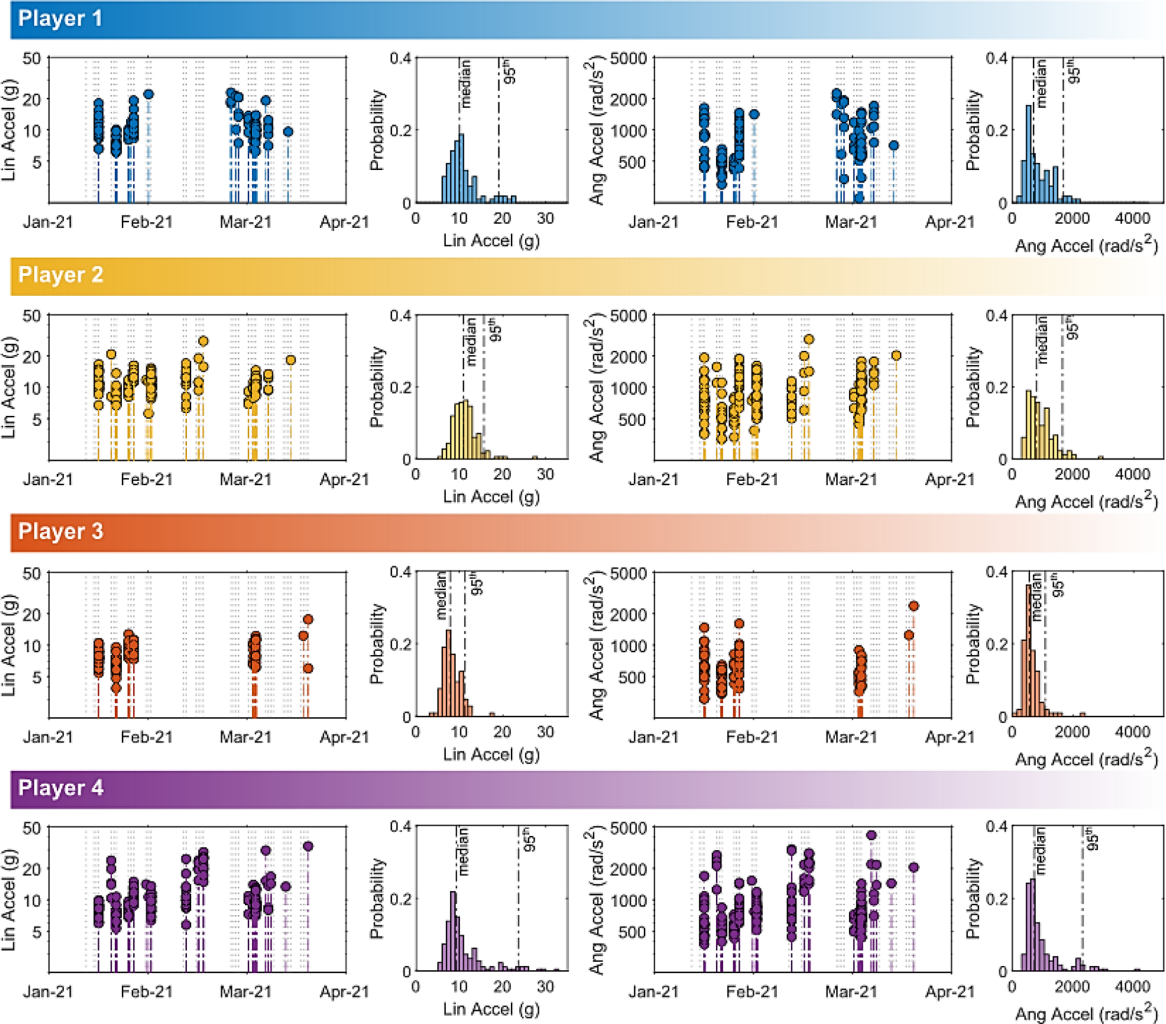
Head kinematics. Here we show the kinematic information and distribution of headers for four individual players. For each player, the peak linear and angular accelerations for headers experienced on each day during the 2021 off-season period are shown in the stem plots, while the peak kinematics distributions are shown in the histograms with median and 95th percentile values indicated by dotted lines.

Over the entire sensor sub-study, the overall sensor failure rate (% days with malfunction) ranged from 2.3 to 23.5% (median = 11.5%) for an athlete and the overall compliance rate (% days wearing sensor) ranged from 5.0 to 41.2% (median = 18.2%), where the compliance rate varied more substantially from period to period (Supplementary Table 3). For the four selected players over the 2021 off-season period with 36 total activity days, sensor failure rate ranged from 0 to 28% (median = 4.7%), player compliance rate ranged from 46 to 100% (median = 86.7%), and device sensitivity (% video headers captured with sensor, when sensor is worn and functional) ranged from 76 to 91%. 227 video-identified headers were not captured by the sensors, and 9513 sensor impacts were not associated with video-identified headers. Our count of false positive impacts is likely an overestimate as it is possible impacts were triggered outside of an athlete’s active playing time. With the sensor-captured data, we evaluated the kinematic distributions of peak linear acceleration (PLA) and peak angular acceleration (PAA) for four players over the period (Fig. 5). Median values for PLA and PAA ranged from 7.9–11.0 g to 555–803 rad/s^2^, respectively. 95th percentile PLA and PAA had greater variation across players with ranges of 11.3–23.6 g and 1088–2314 rad/s^2^, respectively. Most headers from the four players fell within a relatively narrow distribution for both PLA (IQR 8.2–12.1 g) and PAA (IQR: 545–1064 rad/s^2^) with the highest PLA and PAA being 32.7 g and 4183 rad/s^2^, respectively.

## Discussion

To the authors’ knowledge, this is the first heading exposure study to use comprehensive video data across all in-season and off-season periods over multiple calendar years, with over ten thousand video-confirmed headers for longitudinal exposure analysis. The primary goal of the current study was to determine how best to collect and represent longitudinal heading exposure data to study potential associations with long-term structural and functional brain changes. We observe high variance in individual players’ heading exposure, suggesting the need for individualized tracking and assessment of exposure data longitudinally. We also demonstrate the necessity of tracking practices and off-season sessions, which resulted in the highest heading exposure days measured in the study. Finally, despite the availability of wearable head impact sensors to examine exposure, sensors may not fully capture heading exposure due to malfunction, compliance, and thresholding challenges. At the same time, most soccer heading impact kinematics fall within a narrow range of peak head kinematics, suggesting that the number of headers may be sufficient in assessing exposure when sensor data are unavailable.

Using the commonly reported metric of heading rate, the average daily heading exposure for each player is around 2 headers, which is relatively low and consistent with prior studies^18,26,30^. However, this average does not capture the high variance in exposure across players, where each player can have zero exposure on a substantial proportion of days, while daily exposure may be as high as 45 headers. Accumulated over entire seasons or off-season/summer periods, the variance in daily exposure led to high variance in long-term exposure, where some players may sustain as few as 2 headers over a 4-month period, and others may sustain over 200 headers during the same period. Even within the same individual player, we can see from the exposure timelines in Fig. 3 that week-to-week and period-to-period intra-subject variability can also be high, making it difficult to extrapolate exposure findings from a small number of days to long-term estimates, which is very common in existing studies that utilize a cross-sectional study design^31,32^.

With these observations of individual variance, it is expected that any potential heading exposure effects would likely vary across players given high inter-player variations in exposure. As such, it may not be surprising that cohort studies examining brain injury risk of cumulative exposure to soccer headers have yielded inconclusive findings. As shown in our secondary analysis of video analysis methods (Supplementary Table 1), some past studies only analyzed select events, often focusing on games and not practices. Some studies only analyzed sensor-detected impacts, which as we show in the current study may substantially underestimate exposure. There were also studies that used sideline observation as the impact identification method, which could have varying reliability depending on the number of observers and specific protocol used. Furthermore, the high intra-player variability in longitudinal header distribution, where some days or weeks could have frequent and concentrated exposure (Fig. 3), may require additional considerations in cumulative brain injury risk. Future studies should employ accurate longitudinal individual measurements of heading exposure to correlate with individual injury outcomes.

Prior soccer studies typically focused on measuring heading exposure during in-season games^33,34^. In American football, despite head impact rates in practice typically being lower than in games^28,35,36^, total exposure is comparable^37^, and alterations to practices are being investigated to reduce cumulative exposure^38^. We demonstrate that in soccer, practices can account for a greater proportion of a player’s total heading exposure than games and that substantial numbers of headers occur both in the off-season and during the summer. The highest number of headers measured in a single period for a player occurred during a summer off-season period with no official matches. Off-season drill sessions typically focus on skill development, and drills focused on heading training can result in high heading exposure. The presence of contact scenarios associated with relatively higher heading kinematics in practices, particularly in scrimmages, also calls for the inclusion of practices when assessing exposure. As a result, quantification of long-term heading exposure necessitates the inclusion of practice and off-season/summer data.

Comprehensive monitoring of cumulative heading exposure would also require an understanding of impact magnitude in addition to impact frequency^18^. Although wearable head impact sensors are a validated method of measuring impact kinematics^21^ and have seen increasing utility, consistent and complete data collection is limited by player compliance, accessibility, and technical errors especially in longitudinal studies. As shown in our results, noncompliance and sensors malfunction could lead to a substantial number of athletes and days missing sensor data. In our study, most soccer players were not comfortable wearing a mouthpiece sensor during important games for fear of interference with communication and performance. While head impact sensor information would still be valuable to capture when resources are available, missing data need to be accounted for appropriately and sensor data alone may not represent full heading exposure. For soccer headers, as demonstrated by our sensor kinematics dataset, impact kinematics may fall within a tight distribution, which may make it feasible to estimate full heading exposure kinematics with a subsample of headers captured. This is supported by a review paper and Master's thesis, which both suggest that ball deformation during impact modulates acceleration of the head, keeping average peak linear and peak rotational accelerations across studies low and far below concussion range^39,40^. In addition, characterization and quantification of the occurrence of higher-magnitude contact scenarios (e.g., jumping headers) may also help with heading impact kinematics extrapolation. Future studies with a larger kinematics dataset may be needed to develop more rigorous statistical methods for data extrapolation.

While the results of the current study are based on a comprehensive video-analyzed dataset of headers, a few limitations should be considered by researchers when using this study as a framework. First, since we only followed a single university women’s soccer team, our exposure measurements may not generalize to other populations including different age groups, different teams, and across sexes. In fact, we expect greater variance in individual heading exposure when considering these other factors. To quantify variance more broadly, future research should attempt to replicate the current study protocol with different teams. Additionally, while the COVID-19 pandemic and pause in play provided a valuable and unique set of practice-driven data, it resulted in unforeseen factors that contributed additional variance in the study, such as cancellation of some official games and pandemic-related restrictions on in-person activities. Further, while the complete video analysis presented in our study may be a more accurate measure for quantifying full longitudinal exposure, it is time consuming, human resource intensive, and may not be feasible in all situations where exposure metrics are necessitated. If sensor compliance and robustness will be sufficiently improved with further technology development, sensor-guided video verification or sensor-based machine learning methods may help to make exposure estimates more accurate and efficient, as recommended by a recent consensus guideline for on-field wearable devices^19^. Our group has also developed deep learning methods for automated header detection from video, which leverages state-of-the-art computer vision techniques^41^. Such computer vision methods may enable video-based exposure measurements without sensors or extensive human review. However, further validation with on-field data is required^19^. Finally, our study of kinematics included a small sample size of four participants across a single off-season. While we did attempt to deploy mouthpiece sensors as a part of the current study, only a subset of the team participated, confirming the difficulty in obtaining sensor data comprehensively. An analysis of the full heading kinematics dataset from our deployment is summarized in a previous publication^29^. As shown in this previous paper as well as Fig. 5, the head kinematics associated with heading are relatively low compared to those reported from typical sports concussions. While the full exposure estimated using our methodology provides a count of headers, which is a key part of the longitudinal exposure, we recognize that other head impact scenarios (head-head, head-ground) may lead to much higher impact magnitude with acute and long-term injury risk implications. Such scenarios may need to be further examined using a combination of video and sensor methods.

## Conclusion

We recommend that to improve the accuracy of future heading exposure studies and enable correlation with long-term outcomes, assessments should be made on an individual level and include both drills/scrimmages as well as off-season exposure. Full heading exposure should be evaluated using independent video analysis, even when head impact sensor instrumentation is available. While the current study is focused on university varsity women’s soccer, we anticipate that similar individual variance may exist in other sports, and future research may employ a similar methodology to examine this potential variance as well as the need to quantify practice and off-season exposure. If more accurate exposure measurements identify a dose–response relationship between heading exposure and neurological outcomes, individualized monitoring can also be applied to determine and minimize each player’s risk of injury.

### Supplementary Information

Supplementary Information.

## Data Availability

The datasets generated during and/or analysed during the current study are available from the corresponding author on reasonable request.
