# Supplementary material for: Individualized monitoring of longitudinal heading exposure in soccer
Source: Sci Rep. 2024 Jan 20;14:1796. doi: 10.1038/s41598-024-52163-8 (PMC10799858; doi:10.1038/s41598-024-52163-8)
Supplement: Supplementary file 1 — Supplementary Information. [file 41598_2024_52163_MOESM1_ESM.docx]

**Supplemental Information – Definition of Activity Types**

**Game:** a scheduled game of soccer or a session fully simulating a game, involving 10 or 11 players per side. Games in our study included official matches, intersquad matches, playoff matches and exhibition matches.

**Practice:** Any session of play not including games. Typically for player development. Includes drills and scrimmages.

**Drill:** a session involving skill-specific drills. These drills included activities such as passing, juggling, set plays, keep away, attacking and defending, throw-ins and soccer tennis.

**Scrimmage:** a session involving drills that simulated situations that players may encounter during a game but does not fully simulate a game due to field size, player numbers or player positioning. Scrimmages included small-sided scrimmages up to six players per side, and large scrimmages seven to nine players per side.

**Supplemental Table 1 – Secondary Analysis of studies included in McCunn (2020)**

| **Study** | **Video Analysis** | **Method** |  | **Comprehensive Video?** |
| --- | --- | --- | --- | --- |
| Amitay et al 2020 | Yes | Watched all algorithm coded moments that demonstrated potential for header |  | No |
| Andersen (2012) | Yes | Software to quantify, games only |  | No |
| Beaudouin (2020) | Yes | Cross-sectional, select games | | No |
| Caccese (2016) | Yes | Non-comprehensive | | No |
| Lamond (2018) | Yes | Only verifying above threshold impacts | | No |
| Catenaccio (2016) | No | Direct observation | | No |
| Chrisman (2016) | No | Sensor | | No |
| Chrisman (2019) | No | Sensor/direct observation | | No |
| Da Silva(2011) | Yes | SSGs, not comprehensive | | No |
| Forbes (2016) | No | Direct observation | | No |
| Hanlon (2012) | Yes | Only sensor looked at | | No |
| Harriss (2018) | Yes | Used to estimate self-report accuracy | | No |
| Harriss (2019) | Yes | Software + Analyst, games only | | No |
| Harriss (2019) | Yes | Software + Analyst, games only, looked a positions | | No |
| Janda (2010) | No | Direct observation | | No |
| Kaminski (2007) | No | Tally system | | No |
| Kaminski (2008) | No | Tally system | | No |
| Kaminski (2020) | No | Tally system | | No |
| Kelly (2009) | Yes | SSGs they created | | No |
| Koerte (2017) | No | RA Observes | | No |
| Kontos (2011) | No | Observation, cross-sectional | | No |
| Martone (2016) | Yes | Study generated SSGs | | No |
| Miller (2019) | Yes | Comprehensive + Mouthpiece | | Yes |
| Nevins (2018) | Yes | Games only, only sensor impacts reviewed | | No |
| Owen (2014) | Yes | Intervention of making games, sensors as well | | No |
| Patton (2020) | Yes | Video to verify sensor impacts only | | No |
| Press (2017) | Yes | Practices and games + sensors, video only for sensor impacts | | No |
| Rago (2018) | No |  | | No |
| Rahnama (2002) | Yes | Select Games | | No |
| Rich (2019) | Yes | Sensor impacts only visualized in video | | No |
| Rutherford (2009) | No | Observation + self-report | | No |
| Salinas (2009) | No | Observation + self-report | | No |
| Sandmo (2020) | Yes | Video from 10 random matches | | No |
| Sandmo (2019) | Yes | Controlled training protocol (x2), sensor impacts verified | | No |
| Sarajärvi (2020) | Yes | Headers tagged using software | | No |
| Saunders (2020) | Yes | Only sensor impacts verified | | No |
| Stålnacke (2004) | Yes | 2 games, headers estimated for each player | | No |
| Stålnacke (2006) | Yes | 2 games, headers estimated for each player | | No |
| Stephens (2005) | No | Direct observer | | No |
| Stephens (2010) | No | Direct observer | | No |
| Straume-Naesheim (2005) | Yes | Video or observation for four matches to test self-report accuracy | | No |
| Wing (2020) | Yes | Omitted players with less than 20 min, Matches only | | No |

**Supplemental Table 2 – Percentage of sessions with zero headers**

|  | 2019-20 Season | 2019-20 Off-Season | 2020-21 Season | 2020-21 Off-Season | Summer 2021 | 2021-22 Season |
| --- | --- | --- | --- | --- | --- | --- |
| Drills | 84.3% | 71.9% | 78.3% | 49.5% | 74.1% | 80.5% |
| Scrimmage | 84.1% | 82.7% | 66.7% | 75.0% | 71.3% | 83.6% |
| Game | 47.7% | 16.0% | 63.2% | 90.4% | 50.6% | 53.8% |

**Supplemental Table 3 – Sensor error and compliance summary**

|  | 2020-21 Season  (n = 13) | 2020-21 Off-Season  (n = 12) | 2021  Summer  (n = 10) | 2021-22 Season  (n = 11) | Total  (n = 13) |
| --- | --- | --- | --- | --- | --- |
| Percentage Days with Sensor Error for Athletes | | | | | |
| MAX | 22.7% | 41.7% | 33.3% | 15.7% | 23.5% |
| MIN | 0% | 5.6% | 0% | 0% | 2.3% |
| MEDIAN | 0% | 26.2% | 0% | 2.7% | 11.5% |
| Percentage Days Compliant (Sensor Worn by Athletes) | | | | | |
| MAX | 54.3% | 60.0% | 16.7% | 32.9% | 41.2% |
| MIN | 0% | 22.2% | 0% | 0% | 5.0% |
| MEDIAN | 0% | 36.9% | 2.8% | 8.1% | 18.2% |
